# Prevalence and Prognostic Significance of Liver Fibrosis in Patients With Aneurysmal Subarachnoid Hemorrhage

**DOI:** 10.3389/fneur.2022.850405

**Published:** 2022-06-02

**Authors:** Tiangui Li, Peng Wang, Xiao Gong, Weelic Chong, Yang Hai, Chao You, Juan Kang, Fang Fang, Yu Zhang

**Affiliations:** ^1^Department of Neurosurgery, West China Longquan Hospital Sichuan University, Chengdu, China; ^2^Department of Neurosurgery, The First People's Hospital of Longquanyi District, Chengdu, China; ^3^Department of Neurosurgery, Affiliated Hospital of Chengdu University, Chengdu, China; ^4^Department of Ophthalmology, West China Longquan Hospital Sichuan University, Chengdu, China; ^5^Department of Ophthalmology, The First People's Hospital of Longquanyi District, Chengdu, China; ^6^Department of Medical Oncology, Thomas Jefferson University, Philadelphia, PA, United States; ^7^Sidney Kimmel Medical College, Thomas Jefferson University, Philadelphia, PA, United States; ^8^Key Laboratory of Molecular Biology for Infectious Diseases (Ministry of Education), Institute for Viral Hepatitis, Department of Infectious Diseases, The Second Affiliated Hospital, Chongqing Medical University, Chongqing, China; ^9^Department of Neurosurgery, West China Hospital, Sichuan University, Chengdu, China

**Keywords:** intracranial aneurysm, subarachnoid hemorrhage, liver fibrosis, mortality, prognosis

## Abstract

**Objectives:**

To report the prevalence, clinical associations, and prognostic consequences of liver fibrosis in patients with aneurysmal subarachnoid hemorrhage (aSAH).

**Methods:**

In a retrospective study of patients with aSAH, we evaluated three validated liver fibrosis indices and modeled them as continuous-exposure variables, including the aspartate aminotransferase/platelet ratio index (APRI), the fibrosis-4 (FIB-4) index, and the Forns index. The primary outcome was mortality at 90 days. We compared the addition of fibrosis indices to the predictors of the full Subarachnoid Hemorrhage International Trialists model.

**Results:**

A total of 3,722 patients with aSAH were included in the study. According to the APRI, FIB-4 index, and Forns index, 21.7, 17.7, and 11.4% of patients had liver fibrosis. After adjusting for potential confounding factors, liver fibrosis indices were associated with increased 90-day mortality, with odds ratios of 1.35 (95% CI 1.02–1.77) for the FIB-4 index, 1.39 (95% CI.08–1.78) for APRI, and 1.53 (95% CI 1.11–2.12) for the Forns index. Similarly, high liver fibrosis indices were associated with an increased risk of rebleeding. However, the Forns index was not significantly associated with mortality and rebleeding. The addition of FIB-4 indices and APRI into the standard model improved the mortality prediction.

**Conclusions:**

Liver fibrosis is common in patients with aSAH, and high liver fibrosis indices are associated with mortality and rebleeding. The addition of liver fibrosis indices to a standard clinical model significantly improves risk stratification.

## Introduction

In the last decade, aneurysmal subarachnoid hemorrhage (aSAH) had an incidence of 6 to 9 per 100,000 persons per year, with substantial morbidity and a mortality rate of 35% (1). Many studies concluded that hypertension, smoking, and excessive alcohol consumption were risk factors for aSAH (2). Other researchers proved that cirrhosis was associated with an increased risk of hemorrhagic stroke (3). Recently, we found evidence that chronic liver disease was associated with mortality and rebleeding after aSAH (4). These studies highlight the association between advanced liver disease and poor outcomes after aSAH, but it is still uncertain whether there is a similar association between subclinical liver disease and clinical outcomes in patients with aSAH.

Liver fibrosis is a subclinical manifestation of chronic liver diseases, characterized by excessive and abnormal deposition of extracellular matrix components in the liver (5). Previous studies have shown a high prevalence of liver fibrosis in up to 15% of the general population without known liver disease and can be detected using no-ninvasive tests (6, 7).

Liver biopsy is still considered the gold standard for the evaluation of liver fibrosis (8). However, it is expensive, painful, and may be dangerous for patients. Recently, several laboratory indices of liver fibrosis have been reported to be useful predictors of liver fibrosis (9, 10). An elevated fibrosis-4 (FIB-4) index was associated with higher risks of worse outcomes for liver diseases (11). Similar predictive values for adverse liver events were seen in the aspartate aminotransferase/platelet ratio index (APRI) and the Forns index (12, 13).

Liver fibrosis has been correlated with admission hematoma volume, hematoma growth, and mortality in patients with intracerebral hemorrhage (14, 15). For aSAH, the impact of liver fibrosis on rebleeding risks is a significant clinical concern (16). However, its prognostic impact in patients with aSAH is not well known.

We aimed to investigate the prevalence, clinical associations, and prognostic consequences of subclinical liver disease, as defined via three liver fibrosis indices, in patients with aSAH.

## Methods

### Study Design and Data Source

The data that support the findings of this study are available from the corresponding author upon reasonable request. This is a single-center, retrospective cohort study of patients with aSAH. We retrospectively analyzed the data from the electronic medical records of patients with aSAH admitted to West China Hospital, Sichuan University, from January 2009 to June 2019. The ethics committees and the institutional review boards of West China Hospital approved the study and waived the requirement for informed consent.

### Patient Selection

This analysis consisted of patients with aSAH. Aneurysmal subarachnoid hemorrhage was assessed according to neuroimaging (including CT, MRI, or angiography), cerebrospinal fluid analysis, or intraoperatively by a neurosurgeon. Patients were excluded for the following reasons: (1) having overt liver disease and (2) lack of liver fibrosis indices at admission within 24 h. We also excluded the patients whose household registrations were not found in the Household Registration Administration System of Sichuan province. We used personal identification numbers to identify death records from the Household Registration Administration System of Sichuan province. If the identification numbers of patients in the electronic medical record system were wrong or non-existent, or if their household registration was not in Sichuan province, we were unable to find their household registration in the system.

### Clinical Variables

**Figure 1:**
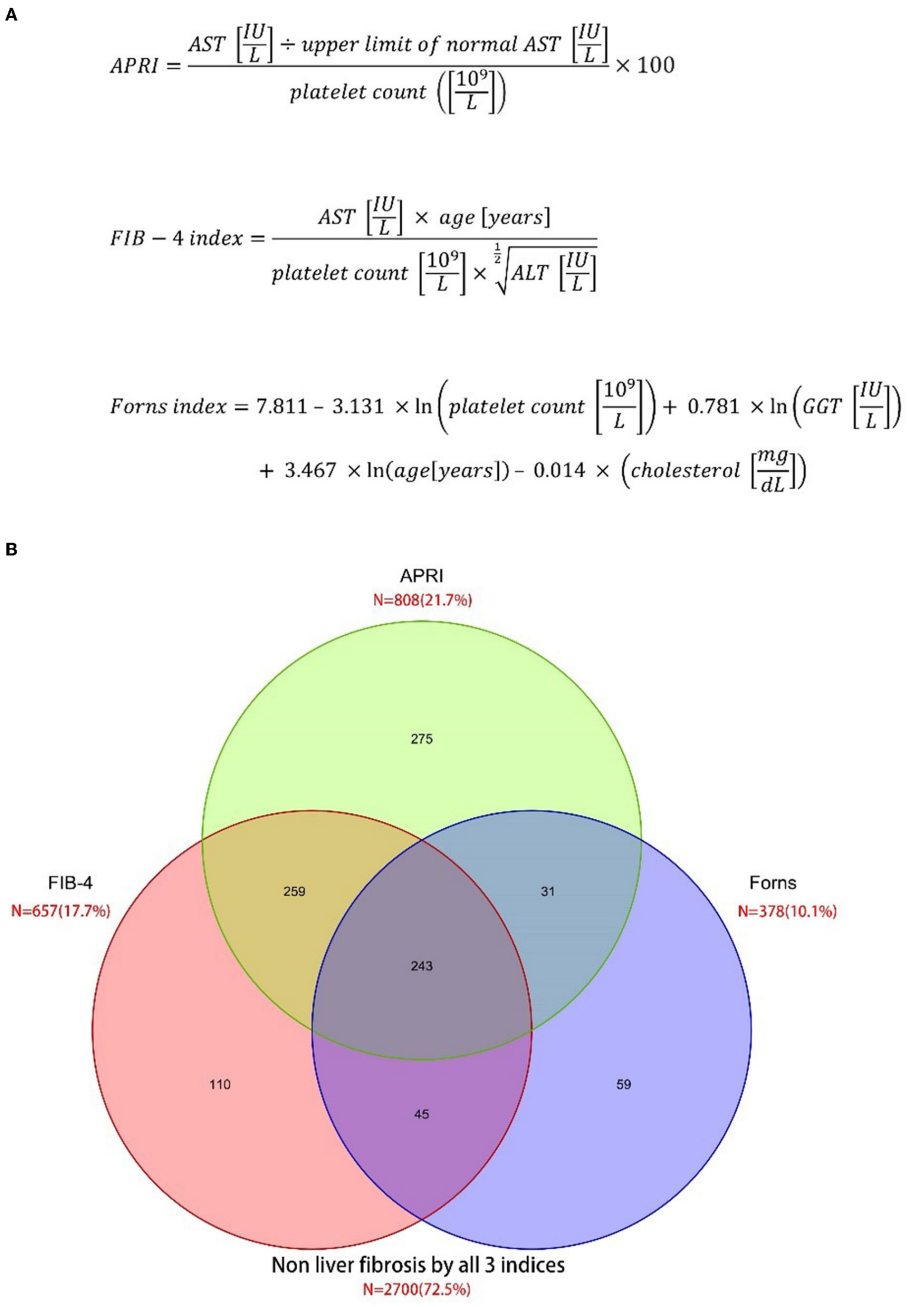
Prevalence of liver fibrosis according to three different scoring systems. **(A)** Three liver fibrosis indices **(B)** venn diagram.

The primary variables were liver fibrosis indices measured at admission. All participants underwent the liver fibrosis assessment using three non-invasive liver fibrosis indices: APRI (17), FIB-4 index (18, 19), and the Forns index (9). Liver fibrosis indices were calculated from the demographic variables and the laboratory data, including aspartate aminotransferase (AST), alanine aminotransferase (ALT), platelet count (PLT), and total cholesterol, which were collected from patients within the first 24 h after admission to the hospital. The formula of the liver fibrosis indices is shown in Figure 1A. We used 3.25 for the FIB-4 index (18, 19), 7 for APRI (17), and 6.9 for the Forns index (9) as cutoffs, according to published literature.

The data of the study population were as follows: demographic variables included age and sex, comorbid conditions (hypertension, diabetes, chronic obstructive pulmonary disease, coronary heart disease, and chronic renal failure), alcohol abuse, smoking, aneurysm characteristics (location and size), external ventricular drain, and aneurysm treatment (clip, coil, and no treatment), and we also collected the Hunt & Hess grade and the Fisher grade at baseline.

### Clinical Outcomes and Definitions

The primary clinical outcome was mortality at 90 days. Other mortality included mortality at 180 days, mortality at 1 year, and mortality at 3 years. We obtained the death records from the Household Registration Administration System database up to 15 August 2021. In China, by law, upon death, the death certificate should be submitted to the household registration office of the Public Security Bureau within 30 days. Because the submission of the death certificate is enforced and the database is always updated, the loss of follow-up in our cohort is almost negligible.

Secondary clinical outcomes were in-hospital complications, including acute kidney injury, pneumonia, hydrocephalus, rebleeding, and delayed cerebral ischemia. Rebleeding was defined as acute worsening in the neurological status along with an increase in hemorrhage volume, which was confirmed in a repeat neuroimaging (CT or MRI scan).

### Statistical Analysis

For all calculations in the statistical analysis, we used software R (version 4.0.3). The data were shown as means ± SD for continuous variables, as medians with the interquartile range, and as numbers (percentage) for categorical variables. All the significance tests were two sided, and *p-*values of <0.05 were considered statistically significant. Multiple imputations have become an appropriate and flexible way to solve missing data, so we repeated all outcomes using multiple regression imputations (20, 21).

According to univariate analyses, the included data were assessed for potential confounding factors. All baseline variables with a *p* value <0.10 in univariate analyses were added into a multivariate regression model. Measured confounders included age and sex, hypertension, diabetes, alcohol abuse, smoking, aneurysm characteristics (location and size), Hunt & Hess grade, Fisher grade, external ventricular drain, and aneurysm treatment (clip, coil, and no treatment).

We assessed improvement in model performance for the addition of liver fibrosis indices to a standard score model by calculating the change in the area under the curve (AUC) and net reclassification improvement (NRI), as recommended by the TRIPOD statement (22, 23). The Subarachnoid Hemorrhage International Trialists (SAHIT) model is a well-established prediction model (24). The SAHIT model was developed from over 10,000 patients and validated in external validation. The full model of SAHIT includes age, neurological grade on admission, history of hypertension, Fisher grade, aneurysm size, aneurysm location, and treatment.

Discrimination was evaluated with AUC curves. A comparison of AUC was performed using the DeLong test. The calibration was evaluated graphically with calibration plots and statistically by computing a goodness-of-fit test of the model using the Hosmer-Lemeshow test.

## Results

A total of 3,722 eligible patients with aSAH were included in the cohort study (Supplementary Figure 1). Among them, 425 (11.2%) were dead at 90 days. According to the APRI, FIB-4 index, and Forns index, 21.7, 17.7, and 10.1% of patients had liver fibrosis (Figure 1B). All liver fibrosis indices were correlated with each other (APRI vs. FIB-4 index: r = 0.71; APRI vs. Forns index: r = 0.62; FIB-4 index vs. Forns index: r = 0.47; all *P* < 0.001). The baseline characteristics of the included patients are described in Table 1. The mean APRI in patients who died was 0.36 and in patients who survived was 1.35 (*p* < 0.001), the mean FIB-4 index in patients who died was 1.65 and in patients who survived was 4.36 (*p* < 0.001), and the mean Forns index in patients who died was 5.51 and in patients who survived was 7.52 (*p* < 0.001), which suggested that all three indices may be prognostic factors for predicting mortality. Other factors that were more common in patients who died included older age, medical history of diabetes, the larger size of the aneurysm, and higher Hunt & Hess grades and Fisher grades.

**Table 1:** Baseline characteristics of the patients.

Characteristics^*^	**Alive** **(*n* = 3297)**	**90-d Dead** **(*n* = 425)**	**P value**
Demographics			
Age, year	54.11 (11.74)	63.41 (9.58)	<0.001
Female	1131 (34.3)	156 (36.7)	0.35
Current smoking	144 (4.4)	25 (5.9)	0.22
Alcohol abuse	641 (19.4)	91 (21.4)	0.37
Medical history			
Hypertension	807 (24.5)	118 (27.8)	0.157
Diabetes	175 (5.3)	42 (9.9)	<0.001
Aneurysm characteristics			
Anterior location	650 (19.7)	85 (20.0)	0.941
Size of aneurysm, cm	0.75 (0.69)	0.75 (0.63)	0.879
Fisher grade			
I	144 (4.4)	13 (3.1)	
II	522 (15.8)	60 (14.1)	<0.001
III	412 (12.5)	42 (9.9)	
IV	1380 (41.9)	229 (53.9)	
Hunt & hess grade			
I	333 (10.1)	29 (6.8)	
II	1752 (53.1)	189 (44.5)	<0.001
III	848 (25.7)	133 (31.3)	
IV	325 (9.9)	66 (15.5)	
V	39 (1.2)	8 (1.9)	
External ventricular drain	68 (2.1)	14 (3.3)	0.146
Operation of aneurysm			
Clip	2299 (69.7)	272 (64.0)	
Coil	404 (12.3)	47 (11.1)	<0.001
No treatment	594 (18.0)	106 (24.9)	
APRI	0.36 (0.15)	1.35 (1.01)	<0.001
FIB-4	1.65 (0.89)	4.36 (3.22)	<0.001
Forns	5.51 (1.64)	7.52 (1.68)	<0.001

* *Mean (SD) or n (%); APRI, Aspartate Aminotransferase/Platelet Ratio Index; FIB-4, Fibrosis-4*.

The logistic regression for the association between liver fibrosis indices and 90-day mortality is summarized in Table 2. In the univariate logistic analysis, all of the three liver fibrosis indices had an association with mortality. After adjusting for all covariates in the model, the multivariate logistic regression analysis still suggested the association between APRI/ FIB-4 index and 90-day mortality, including mortality. The Forns index as binary variables was associated with 90-day mortality in the multivariate logistic analysis, but the association was not significant when the Forns index was input as the continuous variable after logarithmic transformation.

**Table 2:** Unadjusted and adjusted associations between liver fibrosis indices and 90-day mortality.

	**Unadjusted**		**Multivariable Adjustment**	
	**OR (95% CI)**	**p-Value**	**OR (95% CI)**	**p-Value**
FIB−4 index				
Categorical, cutoff 3.25	2.16(1.71–2.72)	<0.001	1.35(1.02–1.77)	0.03
Continuous, log–transformation	5.01(3.42–7.33)	<0.001	2.04(1.25–3.32)	0.004
APRI				
Categorical, cutoff 0.7	1.98(1.60–2.47)	<0.001	1.39(1.08–1.78)	0.009
Continuous, log–transformation	11.66(5.22–26.07)	<0.001	6.72(2.62–17.24)	0.004
Forns index				
Categorical, cutoff 6.9	2.12(1.62–2.77)	<0.001	1.53(1.11–2.12)	0.009
Continuous, log–transformation	5.57(2.54–12.22)	<0.001	1.43(0.55–3.69)	0.47

*APRI, Aspartate Aminotransferase/Platelet Ratio Index; FIB−4, Fibrosis−4*.

The association between liver fibrosis indices as binary variables and other outcomes is summarized in Table 3. The multivariate logistic regression models showed the associations with rebleeding for APRI (odds ratio (OR) 1.69, 95% CI 1.22–2.34), FIB-4 index (OR 1.64, 95% CI 1.14–2.36), and Forns index (OR 1.54, 95% CI 1.01–2.36). None of the liver fibrosis indices were associated with pneumonia, hydrocephalus, and delayed cerebral ischemia.

**Table 3:** Unadjusted and adjusted associations between liver fibrosis indices and outcomes.

		**Multivariable adjustment OR (95%CI)**		
**Outcome**	**Event, *n* (%)**	**FIB−4 cutoff 3.25**	**APRI cutoff 0.7**	**Forns cutoff 6.9**
Mortality at 90 days	411/3722(11%)	1.35(1.02–1.77)	1.39(1.08–1.78)	1.53(1.11–2.12)
Mortality at 180 days	466/3722(12.5)	1.37(1.05–1.78)	1.44(1.13–1.82)	1.57(1.15–2.13)
Mortality at one year	527/3722(14.2)	1.38(1.07–1.77)	1.42(1.13–1.78)	1.58(1.18–2.13)
Mortality at three years	527/3722(14.2)	1.40(1.09–1.79)	1.34(1.07–1.69)	1.65(1.23–2.21)
Acute kidney injury	176/3722(4.7%)	1.43(0.99–2.08)	1.16(0.82–1.66)	1.27(0.82–1.97)
Pneumonia	976/3722(26.2)	1.05(0.85–1.29)	1.02(0.85–1.23)	1.27(1.00–1.63)
Hydrocephalus	391/3722(10.5)	1.00(0.75–1.32)	0.99(0.76–1.29)	1.34(0.96–1.86)
Rebleeding	183/3722(4.9%)	1.64(1.14–2.36)	1.69(1.22–2.34)	1.54(1.01–2.36)
Delayed cerebral ischemia	698/3722(18.8)	1.00(0.81–1.24)	1.19(0.98–1.44)	1.14(0.88–1.49)

*APRI, Aspartate Aminotransferase/Platelet Ratio Index; FIB−4, Fibrosis−4*.

Compared to ARPI and the Forns index, the FIB-4 index outperformed the AUC and NRI at predicting mortality, as was seen by the discrimination index values (Table 4). Using the same predictors as the SAHIT model, our data produced a pooled AUC of 0.79. We added FIB-4, APRI, and Forns indices as predictors into the SAHIT model separately, and including them in the SAHIT model suggested a statistically significant, yet modest, improvement in mortality prediction (*P* = 0.03, 0.01, and 0.02). The Hosmer-Lemeshow test confirmed goodness-of-fit for the SAHIT model (*P* = 0.50, Supplementary Figure 2), SAHIT plus FIB-4 model (*P* = 0.65), SAHIT plus APRI model (*P* = 0.93), and SAHIT plus Forns model (*P* = 0.59).

**Table 4:** Comparative analysis of the discrimination of liver fibrosis indices for 90–day mortality.

	**AUC (95%CI)**	**ΔAUC(P)**	**Categorical NRI, % (P)**
Each liver fibrosis indices			
FIB−4	0.61(0.58–0.64)	NA	NA
APRI	0.56(0.54–0.58)	NA	NA
Forns	0.56(0.53–0.59)	NA	NA
Compartion of liver fibrosis indices			
FIB−4 vs. APRI	NA	0.05(<0.001)	28.5(<0.001)
FIB−4 vs. Forns	NA	0.05(<0.001)	33.7(<0.001)
APRI vs. Forns	NA	0.00(0.89)	0.2(0.97)
Model performance			
SAHIT	0.79(0.77–0.81)	reference	reference
SAHIT +FIB−4	0.79(0.77–0.82)	0.01(0.03)	11.9(0.02)
SAHIT +APRI	0.80(0.77–0.82)	0.01(0.01)	22.6(<0.001)
SAHIT +Forns	0.79(0.77–0.81)	0.00(0.02)	2.4(0.63)

*APRI, Aspartate Aminotransferase/Platelet Ratio Index; FIB−4, Fibrosis−4; SAHIT, full Subarachnoid Hemorrhage International Trialists*.

## Discussion

Liver fibrosis, as defined by existing scores, is common in patients with aSAH. Liver fibrosis indices are associated with mortality and rebleeding. Moreover, the addition of the liver fibrosis indices in a prediction model could accurately predict mortality.

Previous studies confirmed that liver disease was a risk factor for aSAH (4). Liver fibrosis is one of the main manifestations of chronic liver diseases. In recent studies, Parikh et al. found that cirrhosis may be an independent risk factor for aSAH among older individuals. However, they did not assess the predictive value of cirrhosis for significant clinical concerns of aSAH, including rebleeding. Moreover, many other important confounding factors, such as smoking (2), were not adjusted.

To our knowledge, this is the first study to explore the association between liver fibrosis indices and clinical outcomes in patients with aSAH. However, we found several small studies evaluating the association between liver fibrosis indices and poor stroke outcomes. In the study of 432 patients with intracerebral hemorrhage, Parikh et al. (14) explored the association between liver fibrosis indices and mortality. The study revealed that two liver fibrosis indices (APRI and FIB-4) were associated with mortality at 90 days, consistent with our findings; however, they did not explore whether this association remained in long-term outcomes. Baik et al. (25) investigated the association between liver fibrosis and long-term mortality in 395 patients with ischemic stroke. They assessed the degree of liver fibrosis using transient elastography but not liver fibrosis indices and found that liver fibrosis was associated with long-term mortality (median 2.7 years).

The underlying mechanisms of the association of liver fibrosis with mortality in patients with aSAH remain unclear and require further exploration. Hepatic stellate cells play an essential role in liver fibrogenesis and inflammation and are known to be associated with increased circulating levels of several forms of inflammation, endothelial dysfunction, oxidative stress markers, and procoagulant factors (16, 26–28).

This study is the first to investigate the association between liver fibrosis indices and mortality and complications based on patients with aSAH. The key strengths of the study included the large population, long-term follow-up, and comprehensive demographic characteristics. The outcome of patient mortality was based on a provincial population registry, the China Registered Residence system, with accurate records on mortality. We excluded patients with advanced liver disease to better analyze the impact of liver fibrosis indices on clinical outcomes.

Despite these study strengths, the study still had some limitations. First, although we made every effort to acquire data, bias and lack of clinical data are inevitable due to the retrospective cohort study. Second, as we measured liver fibrosis indices only once during admission, it was not possible to assess longitudinal changes in liver fibrosis indices on the risk of death. Third, we could not obtain the cause of death as that was not specified in the registry database.

## Conclusions

Liver fibrosis is common in patients with aSAH. Liver fibrosis indices were associated with all-cause mortality and rebleeding. The inclusion of the liver fibrosis indices led to outcome predictions with superior predictive power compared to the SAHIT score model only. An adequate assessment of liver fibrosis can help improve the prognosis of patients with aSAH.x

## Data Availability Statement

The raw data supporting the conclusions of this article will be made available by the authors, without undue reservation.

## Ethics Statement

The studies involving human participants were reviewed and approved by the Ethics Committee of West China Hospital (No. 20191133). Written informed consent for participation was not required for this study in accordance with the national legislation and the institutional requirements.

## Author Contributions

YZ: study concept. PW, XG, WC, JK, FF, and YZ: acquisition, analysis, or interpretation of data. TL and PW: statistical analysis and drafting of the manuscript. All authors were involved in the design and critical revision of the manuscript for important intellectual content. All authors contributed to the article and approved the submitted version.

## Funding

This work was supported by National Key R&D Program of China (2018YFA0108604), the 1·3·5 project for disciplines of excellence-Clinical Research Incubation Project, West China Hospital, Sichuan University (21HXFH046), the project of Sichuan Science and Technology Bureau (22ZDYF0798), and Clinical Incubation Program of West China Hospital, SCU (2018HXFU008).

## Conflict of Interest

The authors declare that the research was conducted in the absence of any commercial or financial relationships that could be construed as a potential conflict of interest.

## Publisher's Note

All claims expressed in this article are solely those of the authors and do not necessarily represent those of their affiliated organizations, or those of the publisher, the editors and the reviewers. Any product that may be evaluated in this article, or claim that may be made by its manufacturer, is not guaranteed or endorsed by the publisher.
