# Supplementary material for: Prevalence and Prognostic Significance of Liver Fibrosis in Patients With Aneurysmal Subarachnoid Hemorrhage
Source: Front Neurol. 2022 Jun 2;13:850405. doi: 10.3389/fneur.2022.850405 (PMC9201635; doi:10.3389/fneur.2022.850405)
Supplement: Supplementary file 1 [file Data_Sheet_1.docx]

**Supplementary Materials for:**

## Prevalence and Prognostic Significance of Liver Fibrosis Indices in Patients with Aneurysmal Subarachnoid Hemorrhage

eFigure 1 Flow chart of enrollment

eFigure 2 Calibration curves depicting the predicted vs observed one year mortality using the full SAHIT prediction models with and without Liver Fibrosis Indices

#####
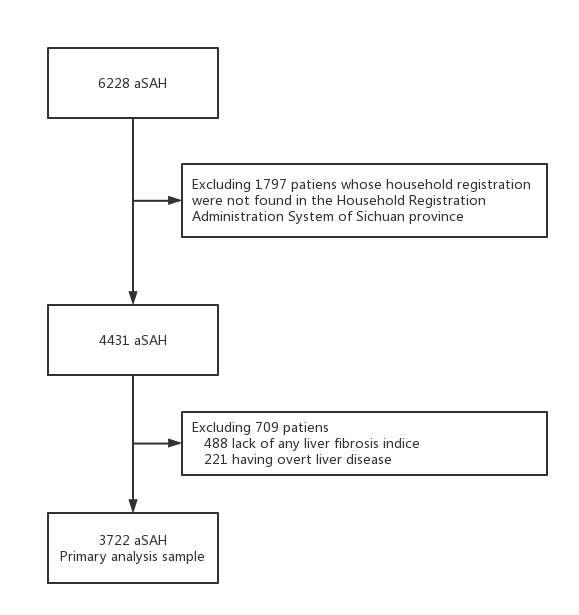
eFigure 1 Flow chart of enrollment


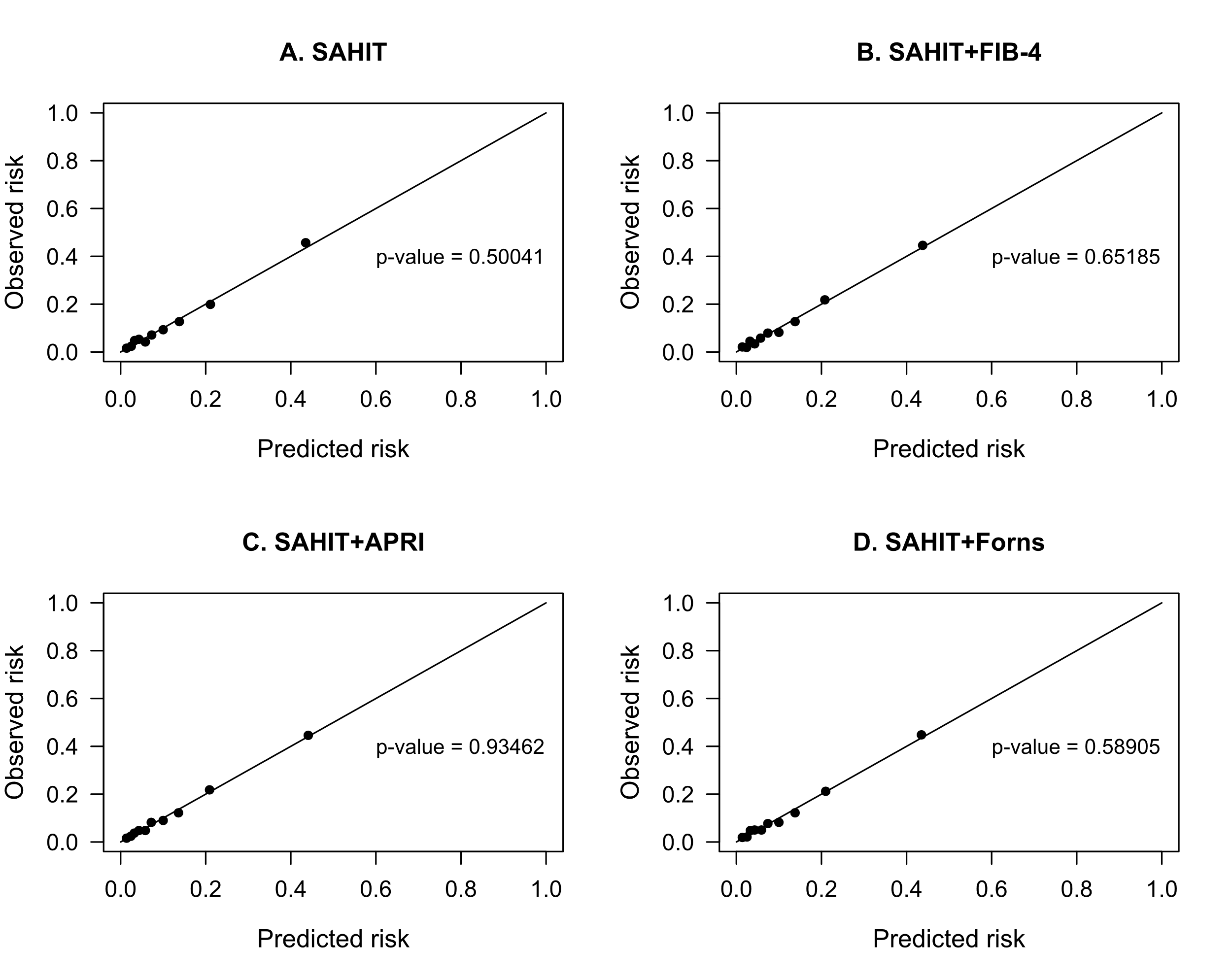


**eFigure 2. Calibration curves depicting the predicted vs observed one year mortality using the full SAHIT prediction models with and without Liver Fibrosis Indices**

SAHIT: Subarachnoid Hemorrhage International Trialists (SAHIT); APRI: Aspartate Aminotransferase/Platelet Ratio Index; FIB-4: Fibrosis-4; SAHIT: full Subarachnoid Hemorrhage International Trialists
